# Supplementary material for: Complementary Feeding Indicators in Relation to Micronutrient Status of Ghanaian Children Aged 6–23 Months: Results from a National Survey
Source: Life (Basel). 2021 Sep 15;11(9):969. doi: 10.3390/life11090969 (PMC8468967; doi:10.3390/life11090969)
Supplement: Supplementary file 1 [file life-11-00969-s001.zip › life-1300468-supplementary.pdf]

Table S1. Proportion of Children Achieving WHO IYCF Indicator by Age Group and Indicator Status among Chil**Table 5.** Adjusted Relative Risk of Anemia, Iron Deficiency, Iron Deficiency Anemia, and Vitamin A Deficiency in Children Aged 6-23 Months, Ghana 2017.

Model	Characteristic	Category	Adjusted Relative Risk [a]	95% CI
Anemia model (n = 372) [b]	Consumed iron-rich foods in past 24 h	Yes	0.61	(0.50, 0.75)
		No	referent	-
	Consumed eggs in past 24 h	Yes	0.54	(0.37, 0.80)
		No	referent	-
	Iron status [b]	Sufficient	0.52	(0.42, 0.65)
		Deficient	referent	-
	Malaria status [c]	Positive	1.74	(1.36, 2.21)
		Negative	referent	-
Iron deficiency model (n = 362) [b]	Currently breastfeeding	Yes	1.58	(1.21, 2.05)
		No	referent	-
	Consumed legumes and/or nuts in past 24 h	Yes	1.26	(1.03, 1.56)
		No	referent	
	Malaria status [c]	Positive	0.26	(0.14, 0.50)
		Negative	referent	-
	Sickle cell status [d]	HbAS, HbSS	0.68	(0.47, 0.98)
		Normal	referent	-
	Child Sex	Male	1.27	(1.05, 1.56)
		Female	referent	-
Iron deficiency anemia model (n = 362) [b]	Currently breastfeeding	Yes	1.53	(1.03, 2.31)
		No	referent	-
	Consumed grains, roots and tubers in past 24 h	Yes	0.66	(0.44, 0.99)
		No	referent	
	Consumed legumes and/or nuts in past 24 h	Yes	1.43	(1.02, 2.02)
		No	referent	
	Malaria status [c]	Positive	0.38	(0.19, 0.74)
		Negative	referent	-
	Wealth quintile	First	referent	
		Second	0.81	(0.55, 1.20)

		Middle	0.59	(0.36, 0.99)
		Fourth	0.63	(0.38, 1.05)
		Highest	0.43	(0.22, 0.82)
Vitamin A deficiency model (n = 398)	Child Sex	Male	1.92	(1.01, 3.65)
		Female	referent	-

Note: All regression models contain child age in months as a continuous variable. [a] Adjusted relative risk calculated using Poisson regression. [b] Anemia defined as hemoglobin <120 g/L; iron deficiency (ID) is defined as BRINDA inflammation-adjusted serum ferritin <12 µg/L; iron deficiency anemia (IDA) is defined as concurrent anemia and ID. Iron deficiency defined as BRINDA inflammation-adjusted serum ferritin concentrations <12 µg/L. [c] Malaria positive defined as current or recent malaria infection by at least on Plasmodium species (i.e., falciparum, vivax, malariae, or ovale). [d] Sickle cell disease and trait represented by HbSS and HbAS, respectively.

Children aged 6–23 months of age, Ghana, 2017

	6–11 Months			12–17 Months			18–23 Months			Total			*p*-Value [c]
	n	% [a]	(95% CI) [b]	*n*	% [a]	(95% CI) [b]	*n*	% [a]	(95% CI) [b]	*n*	% [a]	(95% CI) [b]	
Currently breastfed													
Yes	114	88.0	(77.5, 93.9)	114	75.7	(65.6, 83.5)	57	34.9	(27.7, 43.0)	285	64.8	(57.8, 71.2)	<0.001
No	11	12.0	(6.1, 22.5)	29	24.3	(16.5, 34.4)	88	65.1	(57.0, 72.3)	128	35.2	(28.8, 42.2	
Minimum Dietary Diversity													
Not diverse	99	74.1	(61.5, 83.6)	106	75.8	(65.8, 83.6)	98	67.8	(57.8, 76.3)	303	72.4	(66.3, 77.8)	0.46
Diverse	26	25.9	(16.4, 38.5)	37	24.2	(16.4, 34.2)	47	32.2	(23.7, 42.2)	110	27.6	(22.2, 33.7)	
Minimum Meal Frequency													
Not adequate	58	45.9	(35.8, 56.3)	79	57.0	(48.5, 65.1)	114	80.9	(74.4, 86.1)	251	62.3	(57.1, 67.1)	<0.001
Adequate frequency	67	54.1	(43.7, 64.2)	64	43.0	(34.9, 51.5)	31	19.1	(13.9, 25.6)	162	37.7	(32.9, 42.9)	
Minimum Acceptable Diet													
Not adequate	110	83.3	(70.9, 91.0)	125	89.5	(82.7, 93.8)	131	90.6	(84.1, 94.7)	366	88.1	(83.3, 91.6)	0.26
Adequate diet	15	16.7	(9.0, 29.1)	18	10.5	(6.2, 17.3)	14	9.4	(5.3, 15.9)	47	11.9	(8.4, 16.7)	

Consumption of iron rich food, foods fortified with iron												
Consumed	68	59.7	(50.0, 68.8)	98	68.4	(59.0, 76.5)	120	82.2	(73.5, 88.4)	286	70.8	(65.3, 75.8)	<0.010
Didn't consume	57	40.3	(31.2, 50.0)	45	31.6	(23.5, 41.0)	26	17.8	(11.6, 26.5)	128	29.2	(24.2, 34.7)	
Consumption of vitamin A rich food, foods fortified with intrinsic provitamin A													
Consumed	45	37.9	(28.5, 48.4)	75	53.6	(43.2, 63.7)	89	61.4	(52.5, 69.5)	209	51.8	(45.5, 57.9)	<0.010
Didn't consume	80	62.1	(51.6, 71.5)	68	46.4	(36.3, 56.8)	57	38.6	(30.5, 47.5)	205	48.2	(42.1, 54.5)	

[a] Percentages weighted for unequal probability of selection. [b] CI = confidence interval, calculated taking into account the complex sampling design. [c] *p*-value < 0.05 indicates that the proportion in at least one subgroup is statistically significant

Table S2. Current Breastfeeding, Minimum Dietary Diversity, Minimum Meal Frequency Indicators by Maternal Factors, Children Aged 6–23 Months of Age, Ghana, 2017

	Currently breastfeeding			MDD			MMF		
	n	%[a]	(95% CI)[b]	*n*	%[a]	(95% CI)[b]	*n*	%[a]	(95% CI)[b]
Mother/caregiver is literate		$p = 0.58$ [c]			$p = 0.20$			$p = 0.55$	
Yes	102	57.8	(46.8, 68.1)	102	31.7	(21.5, 44.0)	102	28.2	(21.5, 36.0)
No	61	63.3	(44.0, 79.2)	61	21.7	(12.4, 35.2)	61	33.1	(19.6, 50.1)
Mother/caregiver employment		$p = 0.13$			$p = 0.47$			$p = 0.18$	
No Job	119	67.7	(58.0, 76.1)	119	25.2	(17.1, 35.4)	119	32.9	(25.3, 41.6)
Agricultural or unskilled labor	46	80.6	(65.6, 90.1)	46	35.0	(24.4, 47.4)	46	51.2	(33.4, 68.8)
Skilled labor or professional	74	60.3	(43.2, 75.1)	74	26.0	(15.6, 40.0)	74	34.5	(23.4, 47.7)
Mother/caregiver education status		$p = 0.004$			$p = 0.020$			$p = 0.010$	
Never attended school	74	89.1	(78.6, 94.8)	74	24.7	(16.8, 34.7)	74	54.5	(42.0, 66.5)
Completed primary school or less	45	67.7	(43.9, 84.9)	45	23.9	(13.3, 39.1)	45	33.7	(17.5, 55.0)
Attend or completed JSS	89	61.1	(47.1, 73.5)	89	21.1	(12.1, 34.2)	89	33.5	(25.3, 42.9)
Attended SSS or higher	31	46.0	(29.4, 63.5)	31	50.7	(30.7, 70.4)	31	16.9	(7.4, 34.2)

[a] Percentages weighted for unequal probability of selection. [b] CI = confidence interval, calculated taking into account the complex sampling design. [c] *p*-value < 0.05 indicates that the proportion in at least one subgroup is statistically significant

Table S3. Minimum Acceptable Diet, Iron-Rich Foods, and Vitamin A Rich Foods by Maternal Factors, Children Aged 6–23 Months of Age, Ghana, 2017

	MAD			IRF			VARF		
	n	%[a]	(95% CI)[b]	*n*	%[a]	(95% CI)[b]	*n*	%[a]	(95% CI)[b]
Mother/caregiver is literate		*p = 0.55* [c]			*p = 0.51*			*p = 0.99*	
Yes	102	10	(5.5, 17.5)	102	73.7	(64.0, 81.6)	102	41.1	(32.5, 50.4)
No	61	13.2	(5.9, 26.8)	61	78.5	(62.7, 88.8)	61	41.1	(27.6, 56.1)
Mother/caregiver employment		*p = 0.001*			*p = 0.71*			*p = 0.08*	
No Job	119	6.4	(3.1, 12.8)	119	68.9	(58.6, 77.6)	119	41.0	(31.8, 50.8)
Agricultural or unskilled labor	46	33.3	(22.5, 46.3)	46	74.5	(59.4, 85.4)	46	56.5	(42.3, 69.8)
Skilled labor or professional	74	11.2	(5.2, 22.7)	74	73.3	(60.7, 83.0)	74	35.2	(23.8, 48.5)
Mother/caregiver education status		*p = 0.50*			*p = 0.06*			*p = 0.020*	
Never attended school	74	16.1	(9.0, 27.1)	74	58.5	(46.3, 69.8)	74	42.3	(28.8, 57.0)
Completed primary school or less	45	14.2	(6.2, 29.2)	45	74.8	(55.6, 87.5)	45	44.3	(28.5, 61.4)
Attend or completed JSS	89	11.6	(6.2, 20.6)	89	71.4	(57.6, 82.1)	89	30.2	(19.3, 44.0)
Attended SSS or higher	31	5.8	(1.5, 19.9)	31	88.0	(72.9, 95.2)	31	66.1	(48.9, 79.9)

[a] Percentages weighted for unequal probability of selection. [b] CI = confidence interval, calculated taking into account the complex sampling design. [c] *p*-value < 0.05 indicates that the proportion in at least one subgroup is statistically significant

Table S4. Prevalence of Anemia, Iron Deficiency, Iron Deficiency Anemia, and Vitamin A Deficiency by Anthropometric Indicators among Children Aged 6–23 Months of Age, Ghana, 2017

	Anemia			Iron deficiency			Iron deficiency anemia			Vitamin A deficiency		
	n	%[a]	(95% CI)[b]	*n*	%[a]	(95% CI)[b]	*n*	%[a]	(95% CI)[b]	*n*	%[a]	(95% CI)[b]
Stunting		*p = 0.67* [c]			*p = 0.26*			*p = 0.020*			*p = 0.030*	
Yes	76	47.3	(37.2, 57.6)	76	50.9	(40.5, 61.2)	76	35.8	(25.5, 47.7)	76	4.1	(1.4, 11.2)
No	320	44.6	(37.7, 51.7)	318	43.7	(37.2, 50.3)	319	23.6	(19.1, 28.8)	318	12.0	(8.3, 17.0)
Wasting		*p = 0.53*			*p = 0.28*			*p = 0.61*			*p = 0.63*	
Yes	48	50.7	(33.9, 67.3)	47	53.8	(37.2, 69.6)	48	28.8	(17.5, 43.4)	47	7.9	(2.1, 25.3)
No	345	44.7	(38.1, 51.4)	344	43.6	(37.6, 49.9)	344	25.3	(20.1, 31.3)	344	10.8	(7.5, 15.4)
Underweight		*p = 0.46*			*p = 0.51*			*p = 0.26*			*p = 0.001*	
Yes	78	49.6	(38.5, 60.7)	78	48.8	(36.8, 60.9)	78	31.5	(21.0, 44.2)	78	2.4	(0.9, 6.1)
No	317	44.7	(37.9, 51.7)	315	44.2	(38.0, 50.6)	316	24.8	(19.8, 30.6)	315	12.4	(8.6, 17.4)

[a] Percentages weighted for unequal probability of selection. [b] CI = confidence interval, calculated taking into account the complex sampling design. [c] *p*-value < 0.05 indicates that the proportion in at least one subgroup is statistically significant
